# Low transmission rates of Equine infectious anemia virus (EIAV) in foals born to seropositive feral mares inhabiting the Amazon delta region despite climatic conditions supporting high insect vector populations

**DOI:** 10.1186/s12917-022-03384-4

**Published:** 2022-07-22

**Authors:** Cláudia Fideles Resende, Alison Miranda Santos, Richard Frank Cook, Raphael Mattoso Victor, Rebeca Jéssica Falcão Câmara, Gilberto Pereira Gonçalves, Juliana Gonçalves Lima, André Guimarães Maciel e Silva, Romulo Cerqueira Leite, Jenner Karlisson Pimenta dos Reis

**Affiliations:** 1 Laboratório de Retroviroses, Departamento de Medicina Veterinária Preventiva, Escola de Veterinária, Universidade Federal de Minas Gerais, Belo Horizonte, Minas Gerais 31270-901 Brazil; 2 Faculdade de Medicina Veterinária, Instituto de Medicina Veterinária, Universidade Federal do Pará, Castanhal, Pará 68740-970 Brazil; 3 Department of Veterinary Science, Maxwell H. Gluck Equine Research Center, University of Kentucky, Lexington, Kentucky 40546-0099 USA

**Keywords:** EIAV, Foals transmission, Feral equids, Marajoara breed, Amazon region

## Abstract

**Background:**

Marajó Island, within in the Amazon River Delta, supports numerous bands of feral equids including the genetically distinct Marajoara horses. Approximately 40% of the equids on the island are infected with Equine infectious anemia virus (EIAV). This high seropositivity rate coupled with the need to preserve rare breeds such as the Marajoara horse precludes euthanasia as the primary means for controlling EIAV in this region. In the absence of iatrogenic transmission, spread of this lentivirus is mediated primarily by hematophagous insects, whose year-round prevalence on the island is supported by favorable climatic conditions. In addition, cases of vertical EIAV transmission have been observed suggesting inclusion of seropositive mares in restorative breeding programs could result in their progeny becoming infected with this virus either pre-parturition or post-partum via hematophagous insects. Therefore, the aim of this study was to evaluate EIAV vertical and post-partum insect-mediated transmission rates among foals born to seropositive feral mares until natural weaning. Serum samples from foals born to seropositive feral mares within the Soure municipality, of Marajó Island, were collected to investigate their serological status, using an indirect ELISApgp45, with positive samples confirmed using the classical agar gel immunodiffusion (AGID) assay.

**Results:**

The serological status of 28 foals were monitored over a 2-year period with some subjects, depending on their date of birth, being sampled up to six times. All foals remained with their respective mares until fully weaned at approximately 10 months of age. Only 2 foals (7.14%) in the study group became seropositive against EIAV.

**Conclusion:**

The results demonstrate that in most cases it is possible to obtain seronegative foals born to and eventually weaned by EIA positive mares, even in equatorial regions where substantial rainfall and high temperatures favor the proliferation of insect vectors.

## Background

Marajó Island (Pará state), situated in the Amazon River delta, is the world’s largest fluvial land mass and home to approximately 30,000 equids [1]. Among this equine population are horses directly descended from animals introduced into the region by Portuguese colonizers in the early Eighteenth Century [2]. These imported horses were forced to adapt from the temperate grasslands of Europe to a tropical savanna type environment, where in addition to different plant nutrient sources, they were subjected to harsh hot (30-32 °C) and humid (≥80%) conditions all year round. Moreover, the area has a high annual rainfall (2.800–3.400 mm) with most falling between January and May, resulting in more than two thirds of the Marajó Island landmass becoming inundated during the rainy season [3, 4]. Although many equids on the island are now feral some are still essential for transportation and for the maintenance of cattle and buffalo herds, which is currently the most important economic activity in this region [2, 5].

The initial horse population was interbred with some other Portuguese breeds, including Arabian and Alter Real, and eventually, became recognized as the distinctive Marajoara horse. One of the major characteristics of this breed is adaptation to adverse tropical conditions [2, 6]. Although originally geographically isolated by the island environment, these unique horses have recently been interbred with other horse breeds imported into the area to improve the size, posture and appearance. Unfortunately, most horse breeders have prioritized these phenotypic characteristics, without taking into account the features required for adaptation to the hostile environment and therefore the current interbreeding practices are becoming a major threat to the genetic integrity of the Marajoara horse [2, 7, 8]. The Puruca horse is another highly specialized breed found on Marajó Island that is under an even greater threat from inbreeding programs because the original population comprised of only a comparatively small number of individuals. This breed was generated by breeding Marajoara horses to Shetland ponies and selecting individuals with a maximum standard height of 1.18 m. Modern breeding programs select for larger animals and thereby undermine the major phenotypic characteristic of the breed [2, 7, 8].

Consequently, the equid population of Marajó Island contains distinctive, rare horse breeds that should be preserved, because they possess genetic characteristics uniquely associated with adaptation to the tropical environment of the Amazon River Delta. However, in addition to dilution of these genetic characteristics by interbreeding, another major threat to equids inhabiting Marajó Island is Equine Infectious Anemia Virus (EIAV). In a survey conducted on the island of just 294 equids, 46.26% were found to be seropositive for this virus [9], suggesting it is endemic in the region.

EIAV causes equine infectious anemia (EIA) and is classified within the *Lentivirus* genus of the *Orthoretrovirinae* subfamily in the family *Retroviridae* [10]. The virus is equid specific being reported in horses, donkeys and mules where it produces a persistent infection. Although clinical signs following exposure to EIAV are highly variable, ranging from a subclinical infection to death, in many cases the disease presents in the form of three distinct clinical phases. These consist of an “acute” stage, characterized by pyrexia coupled with thrombocytopenia, a “chronic” phase consisting of recurrent disease episodes, where pyrexia plus thrombocytopenia are associated with anemia, edema, depressed neurological reactions along with cachexia, and finally an “inapparent” phase, where overt disease signs are not observed. Approximately 90% of equids survive the acute and chronic phases, and after a period of 8–12 months, gradually transition to the asymptomatic state, where although blood-associated viral loads are usually low they remain a potential source for viral transmission [11, 12].

In animals subjected to human management, such as working equids, EIAV is often transmitted iatrogenically such as by the reuse of veterinary equipment, especially hypodermic syringes [13–15]. Vertical transmission has also been reported, but the most important means of spread, in the absence of human intervention, is the feeding behavior of large hematophagous insects, including horse flies (Tabanidae family, *Tabanus* or *Hybomitra* genus), deer flies (Tabanidae family, *Chrysops* genus) and perhaps less frequently, stable flies (Muscidae family, *Stomoxys* genus) [16]. Transmission by hematophagous flies does not involve EIAV replication in insect tissues, so the process is purely mechanical. It occurs when feeding on an infected individual is interrupted, causing the fly to seek a second uninfected equid host on which to complete its blood meal. Therefore, the risk of transmission is directly related to the number of infected animals, proximity between animals and density of vectors, the latter being determined by environmental factors, including high temperatures and humidity [15, 17, 18]. These climatic conditions typify the Amazon Delta and so equid populations in this region are subjected to year round high insect vector populations consisting of many different tabanid species [17, 19]. Moreover, during the rainy season flooding can force equid herds to congregate in relatively small areas thereby further increasing the risk of insect mediated EIAV transmission.

In the absence of viable vaccines or other effective treatments, control of EIAV is usually dependent on the identification and removal of infected subjects from the population. The latter is either accomplished by life-long quarantine or more often by euthanasia. Although effective at reducing the overall incidence of EIAV in an equid population, such methods, especially when involving euthanasia, are not compatible with the preservation of rare breeds, where the maintenance of genetic diversity is a major priority. However, it has been found that foals born to and raised by EIAV seropositive mares can remain seronegative [20], suggesting the possibility that virally infected mares can be successfully incorporated into breeding programs. Obviously, if verified, this observation will have significant implications for the preservation of rare or otherwise valuable equids in areas where EIAV is prevalent. The study of McConnico et al. (2000) was conducted in the United States, where high insect vector populations are not continually present and the foals were separated early from their mothers [20]. Therefore, a longitudinal study was conducted under the very different climatic conditions pertaining to Marajó Island where high insect vector populations prevail year round to determine viral transmission rates in foals born to feral, EIAV seropositive mares. Moreover, unlike the North American study [20], mares and their offspring were permitted to remain in close proximity until weaning was naturally complete.

## Methods

### Study area

**Fig. 1:**
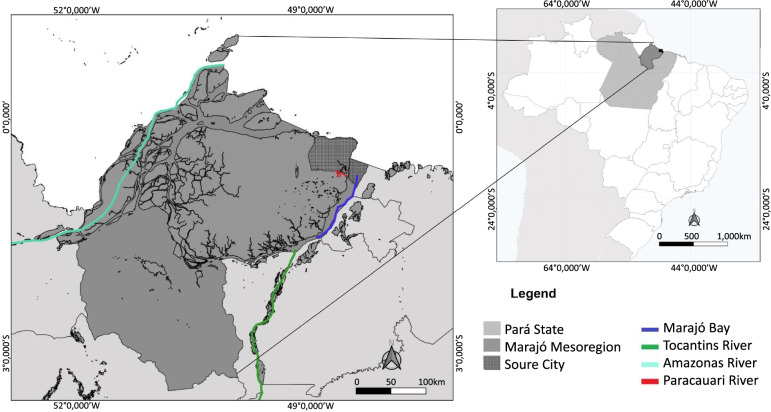
Geographic location of Soure municipality (dark grey) within Marajó Island

The study was performed in a single rural property comprising a mostly native rather than actively managed habitat of about 5000 ha in the municipality of Soure, located on the east coast of Marajó Island. This island is the largest (approximately 49,606 km^2^) member of the Marajó archipelago, and is located in the Amazon River Delta, at the northern boundary of Pará state (Brazil) [21]. Marajó Island is bordered on the west and northwest by the Amazon River mouth, the Atlantic Ocean to the northeast and by the Pará River, a distributary of the Amazon to the east (Fig. 1).

### Samples

The study period was from January 2018 until January 2020 and involved 28 foals (26 horses [*Equus caballus*] and 2 mules [*Equus caballus* x *Equus asinus*]) born to mares that were or became seropositive for EIA. Although the majority of the horse foals were of mixed breed (*n* = 18 or 64.3%), eight subjects (28.6%) were Marajoara horses. Of the 388 equids on the property 75 were used for livestock management while an additional 54 were either in training for livestock management or being prepared for sale. However, the majority (259), including those equids used in this study were not confined to pastures or subject to any form of conventional animal husbandry. Therefore, they were defined as living a “feral” existence, in being free ranging within their environment and, except for sample collection, not subject to regular human interaction, including the administration of vaccines or dietary supplements. Within the feral equid group parturition always occurred under natural conditions and foals remained with their mothers until completely weaned, which normally occurred at approximately 10 months of age.

Blood was collected from animals in the study group by jugular venipuncture into vacuum tubes without anticoagulant and the resultant serum transferred to 1.5 mL microtubes prior to testing. During the course of this longitudinal study foals, depending on birth date, were sampled up to six times to investigate their EIA serological status. Serum samples were stored at -20 °C until tested in the laboratory. All animal experimentation was approved by the Ethics Committee of the Use of Animals of the Universidade Federal de Minas Gerais, under protocol number 171/2018, in accordance with Animal Research: Reporting of In Vivo Experiments (ARRIVE) guidelines.

### Serologic assays

Antibodies to the EIAV gp45 transmembrane envelope glycoprotein were detected by indirect ELISA (ELISApgp45) as described by Naves et al. (2019) [22]. This assay was used as a screening test and previously has been shown to pocess a sensitivity of 98.6% and a specificity of 95.6% when compared to agar gel immunodiffusion (AGID) [22]. Briefly, serum samples were diluted at 1:100 and added (100 μl/well) to 96-well ELISA plates (Nunc-Immuno Plate Maxisorp, USA) previously coated with a synthetic gp45 peptide (0.5 μg/well). All serum samples were analyzed in duplicate. Following a 60 min incubation period at room temperature (RT), each well was washed three times in phosphate buffered saline containing 0.01% Tween 20 (PBS-Tween) prior to the addition (100 μl/well) of a rabbit anti-horse IgG peroxidase conjugate (Sigma-Aldrich, USA) diluted 1:5.000 in PBS-Tween plus nonfat dried milk 0.01%. The ELISA plates were incubated for 60 min at RT and washed 3 times before addition of (100 μl/well) o-phenylenediamine (OPD) substrate (0.5 mg/mL) containing 0.02% of 30 volume hydrogen-peroxide. Color development was permitted for 10 min at RT before the reaction was stopped using sulphuric acid (1 M). Optical density was determined at 492 nm in a microplate reader (BioTek ELx800, USA) and, after normalization based on reading of the positive control, the samples showing optical densities above 0.251 were considered positive.

All ELISApgp45 positive samples were re-tested using a commercially available AGID test kit, according to the manufacturer’s recommendations (Bruch Laboratories, Brazil), to confirm the results. As recommended by the Brazilian regulatory authorities, only serum samples that reacted in both tests were classified as positive [23].

## Results

During the course of this study, blood samples for serological analysis were collected from 26 horse and 2 mule foals. However, the foals used in this study were of different estimated ages, with half of the study group being born after first sample collection date in January 2018. Hence, the foals were divided into three groups. Group A consisted of 14 foals born prior to January 2018 (Table 1), Group B comprised of 8 foals born before the July 2018 collection point (Table 2), while Group C contained 6 foals born prior to the July 2019 sample date (Table 3). At the time of the first sample collection date, foals in Group A ranged in age from 1 to 11 months (Table 1), the 3 oldest (foals 28, 46 and 165 in Table 1) were almost fully weaned and were negatives for EIAV. All the mares in this group, except one (mare of foal 24), were found to be seropositive to EIAV in a survey conducted in January 2018 (Table 1). Three foals in this group produced test positive reactions in both the ELISApgp45 and AGID assays. Foal 230 was found to possess antibodies to EIAV in the sample collected in January 2018, when it was just 1 month old, but gave test negative results at all time points from July 2018 until completion of the study in January 2020. However, foals 45 and 158, that had estimated ages of 7 months in January 2018 and did not have detectable antibodies to EIAV at this first blood sample collection point, were both seropositive in July 2018. Since EIAV causes a persistent infection, these foals were not subjected to further testing. Foal 24 stayed with his mare for a few more months and remained negative until the end of the experiment despite this particular mare becoming test-positive in July 2018.

**Table 1:** Age, breed, sample collection date and results of Group A foals evaluated for EIA

Foal	Breed	Age (months) prior to initial sample date	Date seropositivity of mare confirmed	Month/year of sample collection (foals)											
				Jan/18		Mar/18		Jul/18		Nov/18		Jul/19		Jan/20	
				ELISA	AGID	ELISA	AGID	ELISA	AGID	ELISA	AGID	ELISA	AGID	ELISA	AGID
24	Crossbreed	1	Jul/18	Neg	ND	ND	ND	Neg	ND	Neg	ND	Neg	ND	Neg	ND
28	Crossbreed	10	Jan/18	Neg	ND	ND	ND	Neg	ND	Neg	ND	Neg	ND	Neg	ND
45	Crossbreed	7	Jan/18	Neg	ND	Neg	Neg	**Pos**	**Pos**	ND	ND	ND	ND	ND	ND
46	Crossbreed	11	Jan/18	Neg	ND	ND	ND	Neg	ND	Neg	ND	ND	ND	ND	ND
78	Marajoara	7	Jan/18	Neg	ND	ND	ND	Neg	ND	Neg	ND	Neg	ND	Neg	ND
89	Crossbreed	6	Jan/18	Neg	ND	ND	ND	Neg	ND	Neg	ND	Neg	ND	Neg	ND
137	Crossbreed	7	Jan/18	Neg	ND	ND	ND	Neg	ND	Neg	ND	Neg	ND	Neg	ND
154	Marajoara	8	Jan/18	Neg	ND	ND	ND	Neg	ND	Neg	ND	Neg	ND	Neg	ND
155	Crossbreed	7	Jan/18	Neg	ND	ND	ND	Neg	ND	Neg	ND	Neg	ND	Neg	ND
158^a^	Marajoara	7	Jan/18	**Pos**	Neg	ND	ND	**Pos**	**Pos**	ND	ND	ND	ND	ND	ND
165	Crossbreed	10	Jan/18	Neg	ND	ND	ND	Neg	ND	Neg	ND	Neg	ND	Neg	ND
196	Crossbreed	6	Jan/18	Neg	ND	ND	ND	Neg	ND	Neg	ND	Neg	ND	Neg	ND
200	Crossbreed	6	Jan/18	Neg	ND	ND	ND	Neg	ND	Neg	ND	Neg	ND	ND	ND
230	Crossbreed	1	Jan/18	**Pos**	**Pos**	ND	ND	Neg	ND	Neg	ND	Neg	ND	Neg	ND

Foot notes:

^a^ Foal 158 presented a positive result in ELISApgp45 and negative in AGID in Jan/2018 and in the following test it also became positive in AGID

*ND* Not done

─: Foal had not borned at time of sample collection

**Table 2:** Age, breed, sample collection date and results of Group B foals evaluated for EIA

Foal	Breed	Age (months) prior to initial sample date	Date seropositivity of mare confirmed	Month/year of sample collection (foals)											
				Jan/18		Mar/18		Jul/18		Nov/18		Jul/19		Jan/20	
				ELISA	AGID	ELISA	AGID	ELISA	AGID	ELISA	AGID	ELISA	AGID	ELISA	AGID
398	Marajoara	1	Jan/18	─	─	**Pos**	**Pos**	Neg	ND	Neg	ND	Neg	ND	Neg	ND
404	Marajoara	1	Jan/18	─	─	Neg	ND	Neg	ND	Neg	ND	Neg	ND	Neg	ND
420	Crossbreed	4	Jan/18	─	─	─	─	Neg	ND	Neg	ND	ND	ND	ND	ND
421	Crossbreed	4	Jul/18	─	─	─	─	Neg	ND	Neg	ND	Neg	ND	Neg	ND
422	Crossbreed	1	Jan/18	─	─	─	─	**Pos**	**Pos**	Neg	ND	Neg	ND	Neg	ND
424	Crossbreed	3	Nov/18	─	─	─	─	Neg	ND	Neg	ND	Neg	ND	Neg	ND
436	Crossbreed	3	Jul/18	─	─	─	─	Neg	ND	Neg	ND	ND	ND	ND	ND
439	Crossbreed	2	Nov/18	─	─	─	─	Neg	ND	Neg	ND	Neg	ND	Neg	ND

Foot notes:

*ND* Not done

─: Foal had not borned at time of sample collection

**Table 3:** Age, breed, sample collection date and results of Group C foals evaluated for EIA

Foal	Breed	Age (months) prior to initial sample date	Date seropositivity of mare confirmed	Month/year of sample collection (foals)											
				Jan/18		Mar/18		Jul/18		Nov/18		Jul/19		Jan/20	
				ELISA	AGID	ELISA	AGID	ELISA	AGID	ELISA	AGID	ELISA	AGID	ELISA	AGID
454	Mule	2	Jul/18	─	─	─	─	─	─	**Pos**	**Pos**	Neg	ND	Neg	ND
455	Marajoara	5	Jan/18	─	─	─	─	─	─	Neg	ND	Neg	ND	Neg	ND
516	Marajoara	7	Jul/18	─	─	─	─	─	─	─	─	Neg	ND	Neg	ND
517	Marajoara	4	Jul/18	─	─	─	─	─	─	─	─	Neg	ND	Neg	ND
518	Crossbreed	3	Nov/18	─	─	─	─	─	─	─	─	Neg	ND	Neg	ND
519	Mule	4	Nov/18	─	─	─	─	─	─	─	─	Neg	ND	Neg	ND

Foot notes:

*ND* Not done

─: Foal had not borned at time of sample collection

Two foals in Group B (398 and 422) produced test positive results at only the March 2018 and July 2018 sample collection points (Table 2) respectively. It was estimated that both foals were just approximately 1 month of age at the time of collection and as they were seronegative at all subsequent sample dates. Foals 420 and 436 had to be excluded from further study because they died of non-determined causes after the November 2018 collection point. Mule foal 454 in Group C tested positive in November 2018 when approximately 2 months of age but was seronegative by July 2019 and again in January 2020 (Table 3). These results are similar to those with foals 230, 398 and 422 and once again suggest involvement of maternal antibodies.

## Discussion

The Marajoara and Puruca horse breeds have adapted over centuries to survive in the adverse hot and humid conditions of the Marajó Archipelago. However, while they were originally geographically isolated by their island environment, they have been interbred with many other horse breeds recently introduced into the area. This has resulted in a reduction in the number of individuals possessing the complete compliment of unique genetic characteristics associated with these breeds and has put them at risk of extinction [2, 24]. Furthermore, efforts to preserve both the Marajoara and Puruca breeds are complicated by the fact EIAV is endemic on Marajó Island, hence conventional methods of controlling this virus involving euthanasia of infected individuals are not feasible, especially when the incidence within the region’s equid population probably exceeds 40% [9].

It is important when attempting to preserve an endangered species or breed to maintain as much genetic diversity as possible from the original population, in order to avoid founder effects. These may manifest as undesirable genotypic or phenotypic traits and occur when a new population is established using too few individuals. Consequently, to ensure survival of the Marajoara and Puruca horses in their original form, it may be desirable, or even necessary, to enroll EIAV seropositive mares in any restorative breeding program. However, this would not be possible if there is a high risk of vertical transmission from mare to foal either in utero, during parturition or from the intake of colostrum and breast milk. In human populations infected with the related lentivirus, Human immunodeficiency virus (HIV), for example, it has been demonstrated the risk of vertical transmission from mother to neonate in the absence of antiretroviral drug therapy ranged from 15 to 35% [25]. Moreover, in addition to HIV, it has been shown that other lentiviruses including the Small ruminant lentiviruses (SRLV), Feline immunodeficiency virus (FIV) and several other retroviruses, such as Mouse mammary tumor virus (MMTV), Bovine leukemia virus (BLV) and Human T-cell leukemia virus type 1 (HTLV-1), are all efficiently transmitted via breast milk [26–30].

Although studies in horses are far from exhaustive, it appears that EIAV can be transmitted in utero with an incidence of 100% (7/7) if the mares experience a clinical episode during gestation. On the other hand, the probability of vertical transmission decreases to less than 11% (5/45) if the mare remains asymptomatic. It is important to note that the 11% of foals born to asymptomatic mares did not develop clinical signs until 4 months of age and were maintained in close proximity to an EIAV seropositive foal. Therefore,in these cases transmission may not have originated from the respective mares, but could have occurred horizontally via the infected foal through insect bites [31]. Regardless of this possibility this previous observations [31] clearly suggests the high blood-associated viral loads that are invariably present during febrile episodes [15] significantly increase the probability that EIAV will cross the placenta while if, the infected mare retains its inapparent carrier status, then the risk of viral transmission to the offspring either vertically or via any other route is diminished significantly. In addition, it appears that while milk from EIAV positive mares contains virus that is infectious when inoculated intradermally into seronegative recipient horses, transmission of the equine lentivirus to foals during breastfeeding is not common [32–34]. When taken together, these early studies suggest the risk of vertical transmission in the case of EIAV inapparent carrier mares is significantly lower than in infections with other lentiviruses such as HIV, FIV and SRLV. This prediction is supported by the results of this study and by previous work involving foals born to EIAV positive mares [18, 20, 35, 36]. Indeed, while two foals (45 and 158) became seropositive during the course of this study this occurred when they were somewhere between 7 and 14 months of age, indicating that, vertical transmission is highly unlikely in these cases.

Although vertical transmission of EIAV to foals born to inapparent carrier mares appears to be unusual, they may still be exposed to the virus via the feeding behavior of hematophagous insects, especially as the mare and foal are by definition frequently in very close proximity until weaning is complete. To reduce the risk of insect mediated transmission, Silva et al. (2001) removed foals born to EIAV seropositive mares at 6 rather than 10 months of age and segregated them from the rest of the herd by at least 200 m [18]. In a study reported in the United States 12 foals born to EIAV seropositive mares also remained serogative to this virus although they were separated from their mothers’ at 3–4 months of age [20]. Furhtermore, in this study high insect vector populations were reportedly only present during the summer months [20]. Unfortunately, early weaning results in stress to both mares and their foals, furthermore quarantine measures are resource and management intensive. Thereby, results reported here that most (92.86%) foals remain seronegative to this virus despite remaining with their EIAV seropositive mares throughout the entire weaning process suggest that implementation of such policies is not an absolute requirement for restorative breeding programs. Moreover, in contrast to conditions outlined above in work conducted in the United States [20], equids in this study were feral, living in an environment where EIAV is endemic and where the hot, humid tropical rainforest climate support very high insect vector populations all year round [17, 20, 37].

Collectively, these results demonstrate that of 28 foals studied, just 2 (7.14%) became infected by EIAV. This is in agreement with a survey conducted in the Pantanal Sul-matogrossense region of Brazil, where seropositivity rates in foals approximately 1 year of age were found to be only 3.3%, despite 65.93% of the general equid population being serologically test positive for EIAV [36]. Although in this survey the serological status of each foal’s respective mare was not determined, its findings coupled with results described above suggest foals have a relatively low risk of EIAV infection. Interestingly, this is despite living in an environment that is predicted to be highly favorable for insect mediated transmission. A potential explanation for this might include protective effects from the transfer of EIAV-specific antibodies in colostrum. Initially, these can be present at relatively high concentrations in serum of foals but, decrease over time becoming undetectable by AGID at a mean age of 183 days [20]. Indeed it is highly likely that maternally derived antibodies were responsible for the transitory positive reactions observed in ELISApgp45 and AGID assays on serum samples collected from foals 230, 398, 422 and 454, once they were estimated to be just 1 month old at the time of their positive tests, but were seronegative at all subsequent sample dates.

Although colostral antibodies are usually detectable in serum of foals born to EIAV seropositive mares, to our knowledge, the efficacy of these at preventing EIAV infection has not been investigated. Besides that, another protective factor might be the fact young foals have a more vigorous defensive response to the presence of hematophagous insects than adult equids. These defensive movements often cause the insect to relocate to a more passive host. It has for example been observed that in freely grazing horse herds, foals have only 2.43% the tabanid burden found on older animals [38]. In addition, larger adult animals may be more visually appealing to hematophagous insects and are likely to produce higher concentrations of chemotactic substances, such as carbon dioxide [17].

Although antibody transfer in colostrum and the more aggressive defensive behavior to hematophagous insects may contribute towards the low incidence of EIAV infection reported in this and other studies [20, 36], blood associated viral burdens in inapparent carrier animals is also likely to be extremely important. Unfortunately, this has not been studied extensively, especially in feral equid populations. In experimental situations, clinical episodes of EIA are associated with viral loads that can exceed 10^6^ horse infectious dose _50_ (HID_50_) per mL of peripheral blood. The mouthparts of a large hematophagous insect, such as *Tabanus fuscicostatus*, retain 10 ± 5 nL of blood after an interrupted blood meal that in turn should, in the case of feeding on febrile equids, equate to 5 to 15 infectious EIAV particles. This suggests that, in theory, spread of EIAV can occur following a single fly-bite on an equid with clinical signs of EIA although experimentally it has been shown that transmission by *T. fuscicostatus* under these circumstances occurred in 1 of 7 attempts. In contrast, inapparent carriers have viremia titers of 1 HID_50_ per ml of peripheral blood or less. Under these circumstances, the numerical probability of transmission by *T. fuscicostatus* following an interupted blood meal is approximately 1 in 67,000–200,000 [39–41]. If these numbers apply to the situation in the field, then it is predicted the risk of insect mediated transmission will be low even in situations with high insect vector populations, unless feeding occurs on an equid experiencing clinical signs of EIA. Although further studies are required, results detailed here suggest that inapparent EIAV carrier mares pose only a very low risk for viral transmission to their foals at all stages of life, ranging from in utero development to becoming fully weaned.

## Conclusion

In conclusion, evidence is presented that supports the argument that vertical transmission of EIAV is an infrequent event in foals born to asymptomatic EIAV seropositive mares. Moreover, insect mediated transmission rates from inapparent carrier mares to foals are low thereby, enabling a foal to remain with its mother until fully weaned, even in conditions that are highly conducive for maintaining year-round high density insect vector populations. These findings demonstrate that it is possible to obtain EIAV-free foals, even when born to seropositive mothers providing the mares do not have clinical signs of EIA. This information is important for preserving genetically valuable equid traits, such as those found in the rare Marajoara and Puruca horse breeds that are currently threatened with extinction.

## Data Availability

All data generated or analyzed during this study are included in this published article.

## Abbreviations

AGID: Agar gel immunodiffusion
BLV: Bovine leukemia virus
EIAV: Equine infectious anemia virus
EIA: Equine infectious anemia
ELISA: Enzyme linked immunosorbent assay
FIV: Feline immunodeficiency virus
HID_50_: Horse infectious dose _50_
HIV: Human immunodeficiency virus
HTLV-1: Human T-cell leukemia virus type 1
MMTV: Mouse mammary tumor virus
PBS: Phosphate buffered saline
RT: Room temperature
SRLV: Small ruminant lentiviruses
